# A Study of Valvular Incompetencies in the Lower Limb Veins Using Doppler Ultrasound Findings

**DOI:** 10.7759/cureus.53765

**Published:** 2024-02-07

**Authors:** Sangeetha Arumugam, Durgavajjala Pragnya Manaswini, Prudhvinath A Reddy, Joy A Ghoshal, Nandha Kumar Subbiah

**Affiliations:** 1 Department of Anatomy, All India Institute of Medical Sciences, Mangalagiri, IND; 2 Department of Radiology, All India Institute of Medical Sciences, Mangalagiri, IND

**Keywords:** great and short saphenous veins, perforator veins, venous anatomy, venous duplex ultrasound, varicose

## Abstract

Introduction: Varicose veins (VV) are one of the most common pathologies associated with the venous system of the lower limb. In the Indian population, its incidence is higher, and it is one of the most commonly encountered cases in the hospital. The study aimed to note the widely affected anatomical structure in male and female varicose patients using Doppler ultrasound (DU) examination findings.

Methods: A total of 200 Doppler ultrasound reports of varicose patients were retrospectively analyzed and categorized based on the affected structure. The demographic data of all cases, such as age, sex, brief history, signs, symptoms, and affected side of the lower limb, was noted. Anatomical structures causing venous refluxes in the saphenous systems, junctions, and perforating venous systems were noted. Pearson's correlation coefficient was applied to find out its association.

Results: Out of 200 Doppler reports studied, 133 (67%) were male and 67 (33%) were female patients. The majority, 180 (90%) cases, belonged to C1-C3 stages according to the Clinical, Etiology, Anatomy, and Pathological (CEAP) classification, while 20 (10%) were in C4-C5 stages. Male patients aged between 31 and 40 years were predominantly affected, with their left side being affected most commonly. In female patients, the older age group of 50-60 years was predominantly affected*.* Great saphenous reflux in the groin due to incompetent terminal valve was noted in 126 (63%) cases. In the perforator venous (PV) system, defects in the medial leg perforator (189 (95%)), posterior leg (92 (46%)), and thigh perforator (20 (10%)) were noted.

Conclusion: In the present study, the occurrence of varicose veins is due to the involvement of superficial, deep, and perforator veins with varying percentages. Among the structures, the medial leg perforator was predominantly involved, followed by other perforators. Since most patients were in C1-C3 stages, the involvement of deep veins was noted minimally.

## Introduction

Varicose veins (VV) are one of the most common pathologies associated with the venous system of the lower limb [1]. Varicose veins are part of a broader spectrum of pathologies called chronic venous insufficiency (CVI) [2]. Varicose veins may present as elongated, dilated, and tortuous veins. It causes a broad spectrum of symptoms, such as pain, itch, edema, skin discoloration, bleeding, and ulceration of the affected limb [3]. The incidence of CVI is found to be higher in individuals with a sedentary lifestyle, obesity, prolonged standing, and other comorbid conditions [4]. To understand the pathophysiology and to clinically diagnose varicose veins, it is imperative to understand the anatomy of the venous system of the lower limb.

Many diagnostic and imaging techniques, such as plethysmography, Doppler ultrasound (DU), contrast venography, computed tomography (CT) scans, and magnetic resonance imaging (MRI) scans, are available to evaluate varicose veins [5,6]. In Indian practice, the most common investigations among varicose vein patients are Doppler ultrasound, Duplex color flow imaging, electrocardiogram, and plain ultrasound [7,8]. Of these, Doppler ultrasound is the gold standard technique used to assess varicose veins in a hospital setting.

The current study aims to understand the venous anatomy of varicose veins of the Indian population and correlate with gender and structures affected. The information obtained from the study may be used to improve the diagnosis of varicose veins. It may assist clinicians in selecting a suitable treatment modality for patients based on age group and gender. Also, the rate of various complications and prognosis of the condition can be better predicted with knowledge of the anatomy of varicose veins and their incidences.

## Materials and methods

Study design

This retrospective observational study was conducted from July to December 2022 by the Department of Anatomy and Department of Radiology, All India Institute of Medical Sciences, Mangalagiri.

Study settings

All India Institute of Medical Sciences, Mangalagiri, is a multispecialty tertiary care teaching hospital funded by the Central Government of India.

Ethical clearance

Before the commencement of the study, ethical clearance was obtained from the Institutional Ethical Committee of All India Institute of Medical Sciences (AIIMS/MG/IEC/2022-23/181, dated 08.07.2022).

Data collection tool

A total of 200 Doppler reports were included in the study. Demographic data such as age, sex, brief history of signs, symptoms, and side of the lower limb with varicose veins was noted. Cases with any other vascular abnormality and tumors of the lower extremities were excluded. All patients included in the study underwent B-mode imaging and color flow imaging. They were examined in the supine and standing position while supporting their weight on the contralateral extremity. Both right and left limbs were examined. The Doppler findings of patients with lower limb varicose veins were retrospectively reviewed using the Clinical, Etiology, Anatomy, and Pathological (CEAP) classification. This comprehensive classification system helps clinicians standardize the assessment of chronic venous insufficiency and aids in communication among healthcare professionals. The classification encompasses a range of venous disorders, from simple telangiectasias (C0) to severe venous ulcers (C6), considering clinical signs, symptoms, underlying causes, anatomical involvement, and the physiological impact of the venous disorder [1,2]. Based on the anatomy component of the CEAP classification, superficial, deep, and perforator veins were studied. Venous reflux from deep to superficial at various levels and interrelations between the saphenous system and the perforator venous (PV) system were noted. Valvular incompetence was assessed in the sapheno-femoral junction (SFJ), sapheno-popliteal junctions (SPJ), and other perforator veins.

Data analysis 

The Statistical Package for Social Sciences (SPSS) software (IBM SPSS Statistics, Armonk, NY) was used to analyze the data obtained. Descriptive statistics were used to clean and describe the data before entering it into SPSS. Frequency/percentages were used to specify categorical variables such as age and gender. The relationship between the anatomical structures affected and the clinical severity of the disease was studied using Pearson's correlation coefficient.

## Results

A total of 200 reports were retrospectively analyzed, and inferences were drawn. Of the 200 reports studied, 133 (67%) belonged to male patients, and 67 (33%) were female. The patients included in the study were between 25 and 75 years old. The age distribution of male and female patients is shown in Table 1.

**Table 1:** Age- and gender-based distribution of patients

Age group	Male (n=133)	Female (n=67)	Total (N=200)
21-30 years	9	5	14
31-40 years	37	14	51
41-50 years	30	20	50
51-60 years	33	17	50
61-80 years	24	11	35

The majority of the affected males were in the age group of 31-60 years, while affected females were between 41 and 50 years. The mean age of the patients examined was 46.4 years. According to CEAP, the patients were classified into mild to moderate group (C1-C3) and severe group (C4-C6). Among the 200 cases, 180 (90%) belonged to the C1-C3 category, and the remaining were in the C4-C5 category.

Structures affected in groin reflux

Saphenous venous reflux at the groin was noted in 126 (63%) limbs. The terminal valve at the SFJ was affected in 84 male and 42 female limbs (Table 2).

**Table 2:** Distribution of structures affected in males and females SFJ: sapheno-femoral junction, SPJ: sapheno-popliteal junction

Structures affected	Male	Female	Total (N=200)
SFJ incompetency	84	42	126 (63%)
Thigh perforator	16	4	20 (10%)
Medial leg perforator	123	66	189 (95%)
Lateral leg perforator	17	9	26 (13%)
Posterior leg perforator	60	32	92 (46%)
SPJ defect	37	22	59 (30%)
Deep vein defect	30	10	40 (20%)

Reflux in the popliteal fossa

SPJ reflux was noted in 10 (5%) limbs and caused the small saphenous vein (SSV) reflux in the popliteal fossa (Table 2).

Perforator reflux

**Figure 1:**
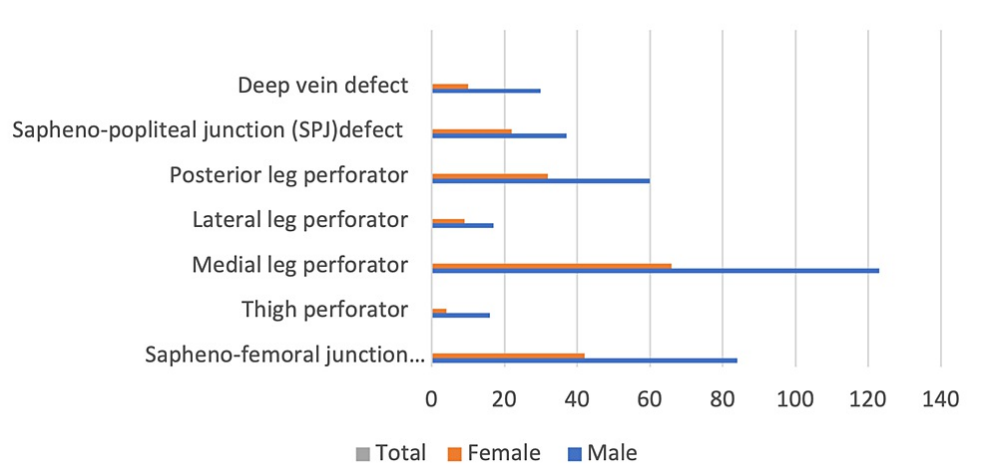
Frequency of distribution of structures involved in venous insufficiency X-axis: total number of cases affected, Y-axis: various structures involved SPJ: sapheno-popliteal junction

Saphenous venous reflux caused by defects in the PV system was noted in 189 (95%) limbs. Among the perforators affected, 20 limbs had defects in the thigh perforator, 189 in the medial leg perforator, 23 in the lateral leg perforator, and 92 in the posterior leg perforator (PLP) as shown in Figure 1.

**Figure 2:**
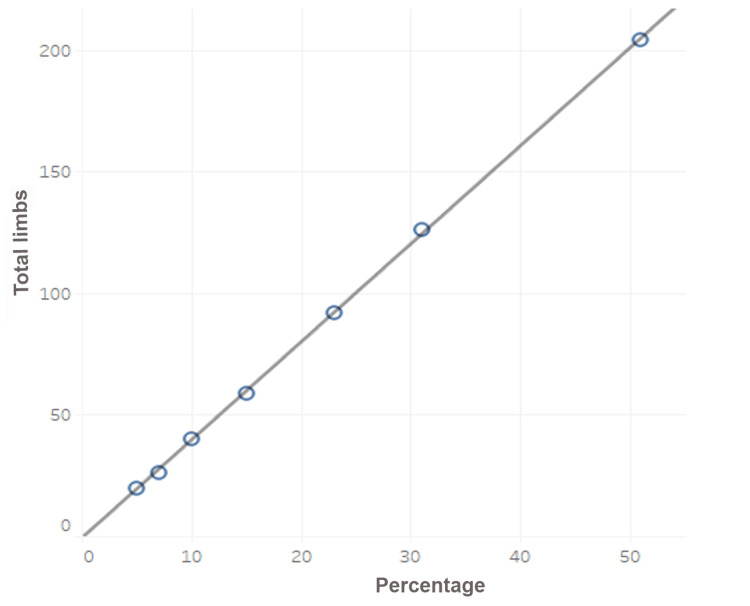
Correlation between perforator incompetence and total limbs affected X-axis: percentage of perforator incompetence, Y-axis: total limbs affected

The Pearson's correlation coefficient is r=0.9, suggestive of the strong association between perforator involvement and varicose affected (Figure 2).

## Discussion

The prevalence of varicose veins shows regional and gender variations [7]. In India, the prevalence of varicose veins (VV) in the general population is 10% irrespective of their gender and occupational history [8]. Earlier studies done in the Indian population have shown that varicose veins were predominantly seen in males in their middle age between 21 and 40 years [8,9]. Findings in the present study showed that 150 (75%) affected individuals were in the age group of 30-60 years, 36 (18%) above 60 years, and 14 (7%) under 30 years. The present study shows that the least prevalence is seen in individuals under 30 years old, which could be attributed to healthy lifestyle changes and earlier interventions. In the present study, there were 133 (67%) males with VV compared to 67 (33%) females with VV. This gender difference could possibly be because male patients primarily seek medical support compared to female patients in the Indian setup [8,9]. On the contrary, few studies in Western countries have shown that VV is more prevalent in female patients, which could be due to awareness about varicose veins, their complications, cosmetic reasons, and financial affordability [6,7].

Regarding the side of the limb affected, involvement of the left extremity was more in males compared to females, which corresponds to earlier Indian studies [8,9]. The anatomical basis of left-side involvement is attributed to the extrinsic compression of the left common iliac vein at the convexity of the sacroiliac joint by the right common iliac artery [6].

Venous reflux is defined as a point through which blood passes from the deep to the superficial system, causing varicosity [10,11]. Significant refluxes are inguinal reflux, popliteal reflux, and perforator reflux. In the present study, saphenous reflux at the SFJ due to saphenofemoral valve defect was observed in 126 (63%) limbs examined belonging to both genders. Short saphenous vein (SSV) reflux due to SPJ incompetency was noted in 60 (30%) limbs in the present study, which is higher than in earlier studies. According to earlier studies, the frequency of reflux in the SPJ was between 9.4% and 28% [10,11].

Another most common factor for saphenous reflux is perforator incompetency. There are multiple perforators along the length of the great saphenous vein (GSV) and SSV. These perforators are grouped as thigh and leg perforators. Leg perforators are subdivided into medial (upper, middle, and lower), posterior, and lateral leg perforators. Normally, valves of the perforators allow the unidirectional flow of blood from superficial to deep veins [1,12]. Incompetency of perforator valves causes retrograde blood flow into the superficial veins, causing reflux.

Moreover, the failure of check valves in the perforating veins directs blood flow into the unsupported superficial veins due to the pressure generated in the deep veins by muscular contraction and other local factors [13,14]. In the current study, 189 (95%) medial leg perforators were affected in both male and female individuals. Incompetency of the medial leg perforator of the left limb was more significant in male patients compared to other perforators. This suggests that the incompetency of the medial leg perforator needs to be carefully evaluated. In correlation to this finding, earlier studies have also reported the predominant involvement of medial leg perforators, about 70%-90% [15]. Next to the medial leg perforator, posterior leg perforator (PLP) incompetency was seen in 92 (46%) individuals. SSV joins with the popliteal vein at the SPJ, which ascends into the thigh and continues to the femoral vein. Together, SPJ incompetency and the posterior leg perforator defect could cause SSV reflux. Lateral leg perforator incompetency was observed only in 26 (13%) cases. In the present study, isolated perforator involvement was low, with more cases showing multiple perforator involvement. The present study's findings suggest that duplex examination of the medial leg perforator is most important in all individuals with primary varicose veins before surgical intervention.

The prevalence of deep venous reflux in this study was seen only in 40 (20%) limbs, and it was noted in the posterior calf region. The reduced number of deep veins affected in the current study could be due to the earlier stage presentation of the cases, mainly in the C1-C3 category according to the CEAP classification. Similarly, a study done in the Thailand population also reported less prevalence of deep venous reflux in primary cases compared to secondary and post-thrombotic complications in the late stages of venous insufficiency [16]. Other studies also reported variability in the prevalence of deep venous reflux in C1-C3 clinical cases. Hence, multifactorial components exist in cases with primary varicose veins, and it is difficult to determine the relative contribution of each component to varicose veins.

Limitations of the study

The present study was conducted using 200 Doppler report findings in patients belonging to a particular region in South India. Other contributing risk factors and pathogenesis of varicose veins were not evaluated and correlated. Hence, the initial findings of the present study need to be further validated with a larger sample size.

## Conclusions

In the present study, there is involvement of superficial, deep, and perforator veins with varying percentages. Superficial veins are involved secondary to valvular and perforator incompetence. Medial leg perforator incompetency was observed predominantly. The low prevalence of deep venous reflux and SPJ reflux in the current series suggests that Doppler examination of the popliteal fossa and deep veins can be skipped in earlier stages of varicose veins.
